# Supplementary figures and images for: A mixed-model approach for estimating drivers of microbiota community composition and differential taxonomic abundance
Source: mSystems. 2023 Jul 25;8(4):e00040-23. doi: 10.1128/msystems.00040-23 (PMC10469806; doi:10.1128/msystems.00040-23)

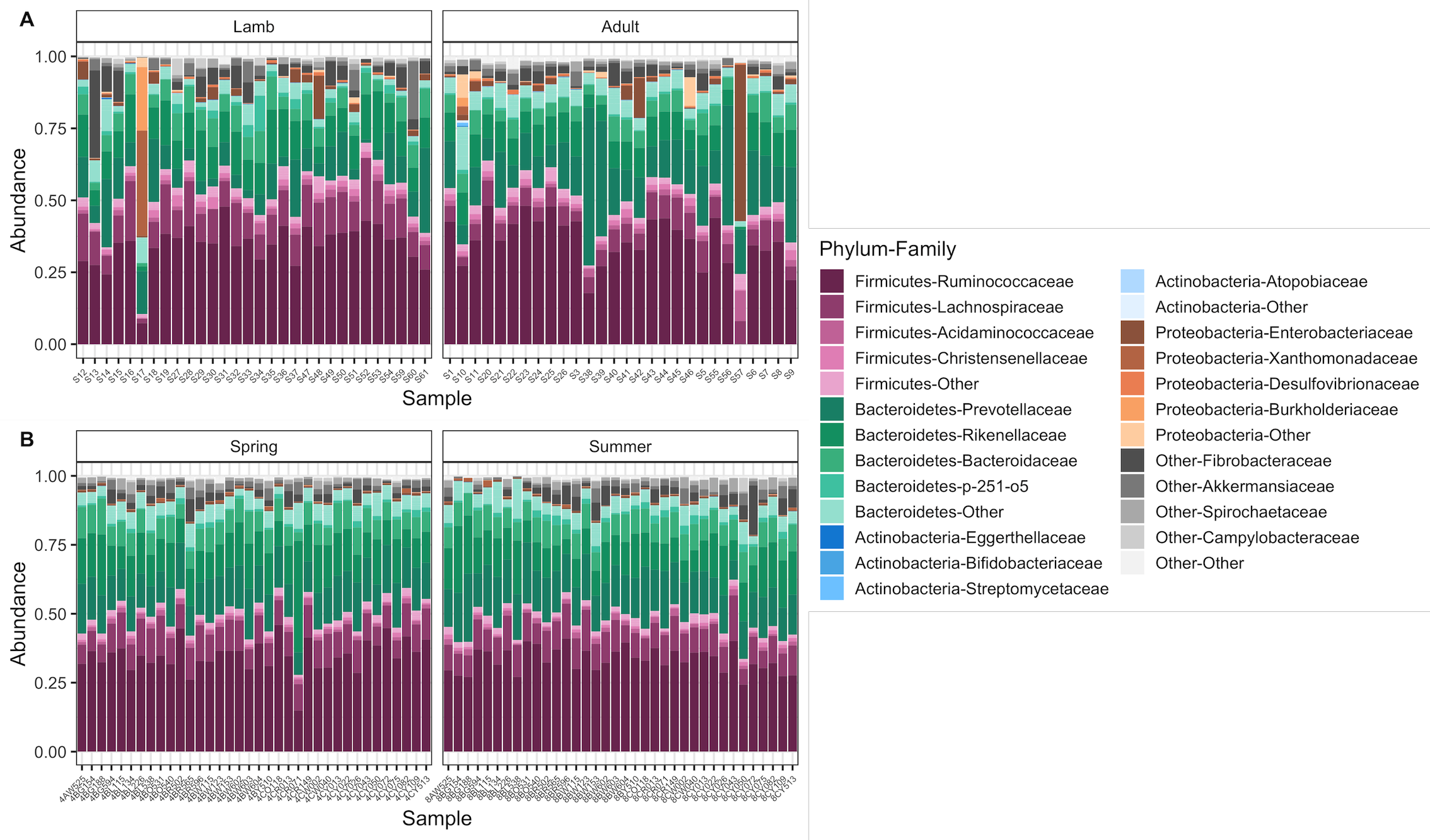

Supplement: Figure S1 — Relative abundance of phyla and families in example data sets. [file msystems.00040-23-s0001.tif]

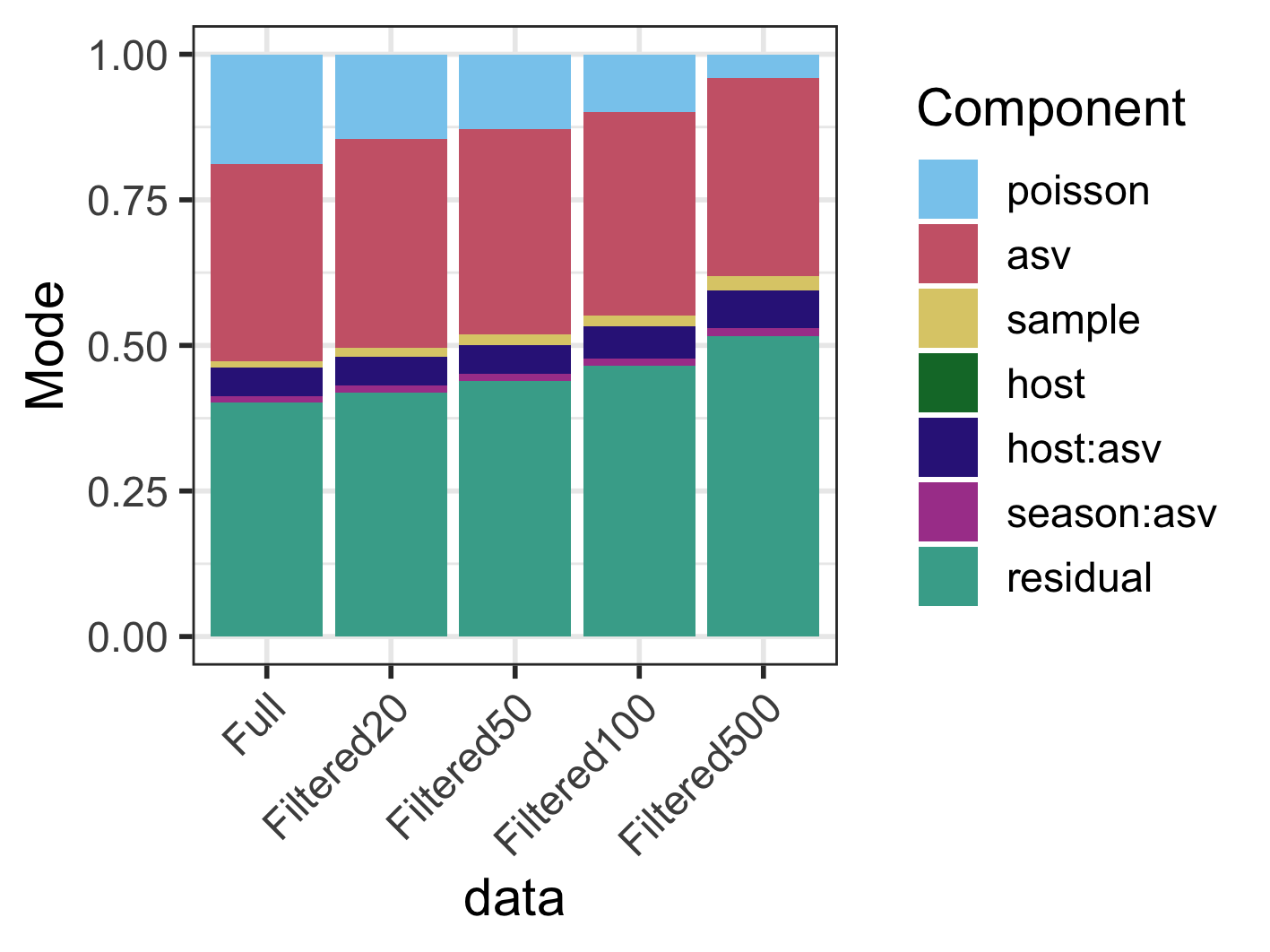

Supplement: Figure S2 — Proportion variance across multiple levels of initial abundance filtering. [file msystems.00040-23-s0002.tif]

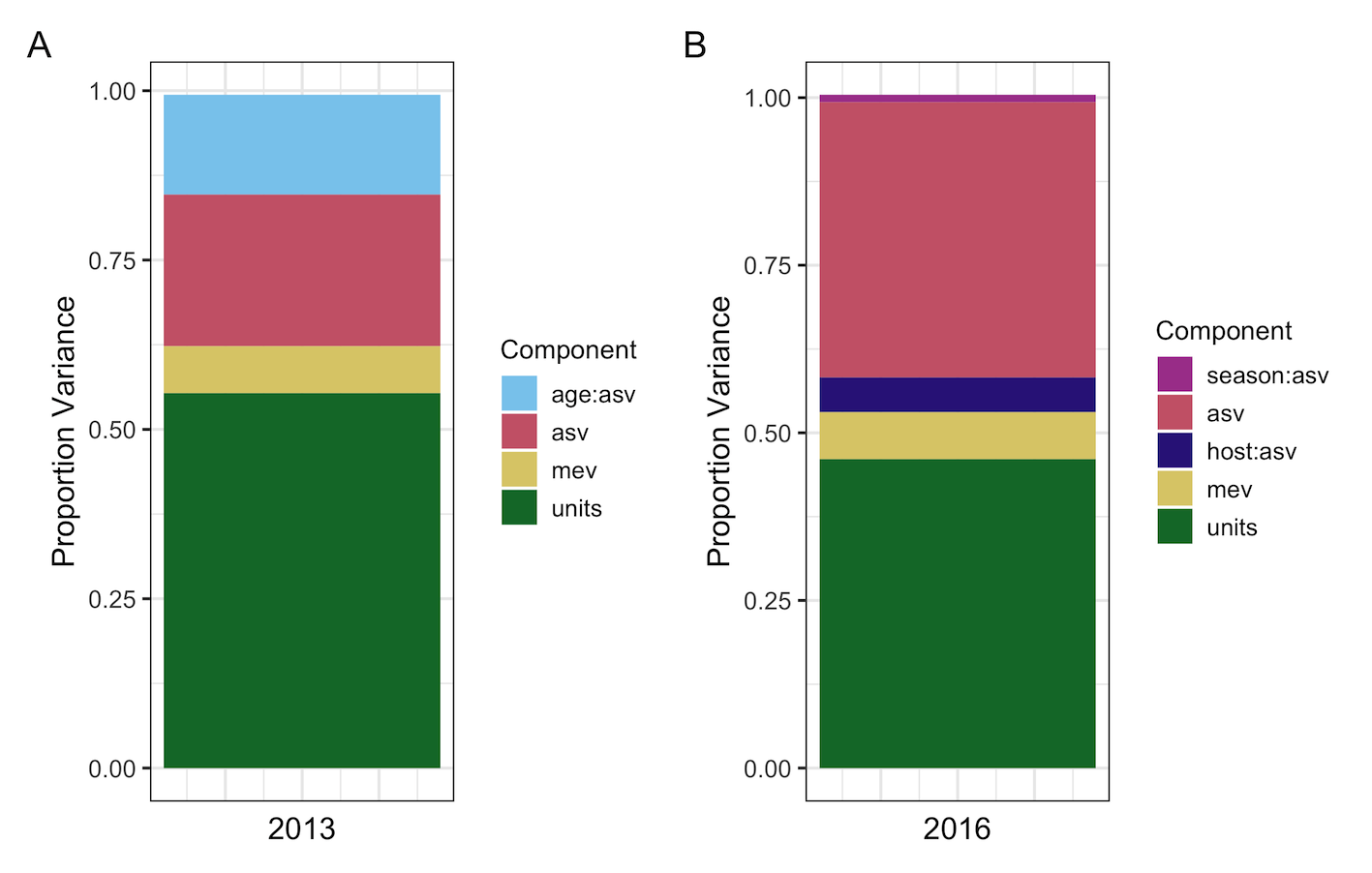

Supplement: Figure S3 — Proportion variance for CLR models. [file msystems.00040-23-s0003.tif]

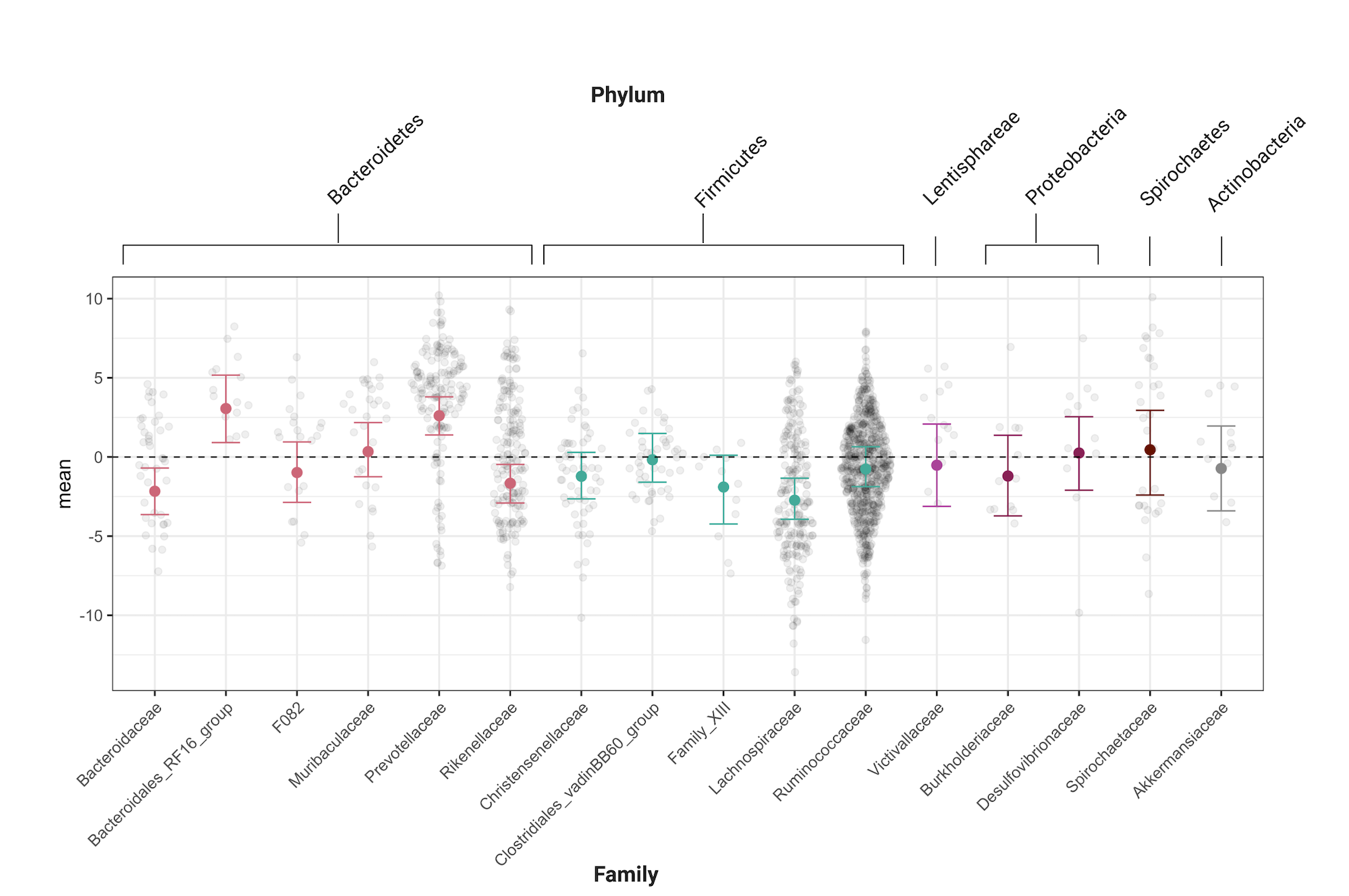

Supplement: Figure S4 — Differential abundances across age classes accounting for multiple levels of taxonomy. [file msystems.00040-23-s0004.tif]
